# Biomarker Identification for Alzheimer’s Disease Using a Multi-Filter Gene Selection Approach

**DOI:** 10.3390/ijms26051816

**Published:** 2025-02-20

**Authors:** Elnaz Pashaei, Elham Pashaei, Nizamettin Aydin

**Affiliations:** 1 Department of Medical and Molecular Genetics, Indiana University School of Medicine, Indianapolis, IN 46202, USA; elhpasha@iu.edu; 2 Department of Computer Engineering, Faculty of Computer and Informatics Engineering, Istanbul Technical University, Istanbul 34467, Türkiye; naydin@itu.edu.tr

**Keywords:** Alzheimer’s disease (AD), multi-filter gene selection, machine learning in AD, biomarkers, protein–protein interaction (PPI) network, hub genes in AD, random forest (RF)

## Abstract

There is still a lack of effective therapies for Alzheimer’s disease (AD), the leading cause of dementia and cognitive decline. Identifying reliable biomarkers and therapeutic targets is crucial for advancing AD research. In this study, we developed an aggregative multi-filter gene selection approach to identify AD biomarkers. This method integrates hub gene ranking techniques, such as degree and bottleneck, with feature selection algorithms, including Random Forest and Double Input Symmetrical Relevance, and applies ranking aggregation to improve accuracy and robustness. Five publicly available AD-related microarray datasets (GSE48350, GSE36980, GSE132903, GSE118553, and GSE5281), covering diverse brain regions like the hippocampus and frontal cortex, were analyzed, yielding 803 overlapping differentially expressed genes from 464 AD and 492 normal cases. An independent dataset (GSE109887) was used for external validation. The approach identified 50 prioritized genes, achieving an AUC of 86.8 in logistic regression on the validation dataset, highlighting their predictive value. Pathway analysis revealed involvement in critical biological processes such as synaptic vesicle cycles, neurodegeneration, and cognitive function. These findings provide insights into potential therapeutic targets for AD.

## 1. Introduction

Alzheimer’s disease (AD) is a progressive brain disorder, primarily affecting older adults, which slowly destroys memory and cognitive functions [1,2]. It causes progressive loss of brain cells that makes the brain shrink and performance decline [3]. Although no one is yet sure what triggers AD, two of the main pathological hallmarks in a brain suffering from the disease—tau tangles and beta-amyloid plaques—are generally believed to be involved in its processes [4,5]. While beta-amyloid aggregates form plaques that interfere with neuronal communication, tau tangles predominantly affect brain regions associated with memory [6]. The loss of communication ultimately causes neurons to degenerate. Chronic neuroinflammation has also been associated with promoting AD via the increased activity of protective immune cells (microglia and astrocytes) that lead to greater neuronal damage [7,8,9].

Alzheimer’s disease constitutes a profound affliction for individuals and represents an escalating public health emergency. As of 2023, an estimated 6.9 million Americans aged 65 and older are living with Alzheimer’s dementia, with projections estimating this number could grow to 13.8 million by 2060 without significant medical breakthroughs. Alzheimer’s remains the seventh-leading cause of death in the United States, with 119,399 deaths recorded in 2021. Between 2000 and 2021, reported deaths from AD increased by more than 140%, highlighting its increasing burden on individuals, families, and society. These alarming statistics underscore the urgency for more research and improved care strategies for those affected by Alzheimer’s disease [10].

Even though novel drugs are under investigation, so far, no agent has been found capable of preventing or reversing AD progression [11]. Existing drugs such as Acetylcholinesterase inhibitors and N-methyl-D-aspartate (NMDA) receptor antagonists [12] offer only modest symptomatic relief, thus underscoring an urgent need for innovative and practical strategies.

One of the most promising strategies in the fight against AD is the identification of biomarkers that enable early diagnosis, monitor disease progression, and reveal therapeutic targets [1,3]. Biomarkers are also valuable for understanding the mechanisms of AD and assessing the efficacy of treatments. Advancing biomarker discovery could enhance targeted therapies, especially in areas where AD treatment options are limited [13].

There are several methods for identifying biomarkers for AD, each offering unique insights. Differential analysis is one of the common methods used in research; it identifies genes with a significant difference in expression level between normal and diseased samples. This technique became an essential approach for identifying possible biomarkers, as described in [14,15,16], using DEGs (differentially expressed genes) to identify possible key AD-related biomarkers. Another way to look at how genes interact with each other in biological systems is through gene interaction networks, like Protein–Protein Interaction (PPI) networks [17]. For example, Cao et al. (2024) [18] used a PPI network to identify important genes, such as HDAC7, KAT5, and SIRT2, as potential AD biomarkers.

More recently, machine learning (ML) and deep learning (DL) models have been employed to analyze complex data and improve biomarker identification [19,20]. For instance, Alamro et al. (2023) [21] used ML and other techniques such as feature selection to identify hub genes associated with AD. Their report found that five specific genes, which were found through LASSO and Ridge regression, could successfully detect AD cases from controls, reaching an area under the curve (AUC) of 0.979. This study also shows the prospect of advanced ML models to improve biomarker findings.

Aside from the traditional genetics and ML approaches, newly developed technologies such as single-cell sequencing [22], spatial transcriptomics [23], and Ribonucleic acid (RNA)-related biomarkers [24] have turned out to be a major cornerstone in the identification of AD biomarkers [25]. Single-cell level sequencing allows one to inspect the interaction of individual cells and their dynamics, bringing in different responses and changes that are typical for the understanding of such complex diseases as AD. For instance, Mathys et al. (2024) [26] used this method to study 1.3 million cells obtained from the brains of deceased individuals, identifying vulnerable neurons in Alzheimer’s disease. They also demonstrated the involvement of the Reelin signaling pathway. In this sense, Spatial Transcriptomics provides an extra step by way of gene data related to tissues that will show and tell a story about the distribution of gene activity in tissue [23,27]. The method is effective for identifying AD-affected spots in the brain as well as in the detection of potential markers that guide the course of the disease [27]. For example, Fan and Li [28] used Spatial Transcriptomics in combination with single-cell RNA sequencing to investigate AD in the prefrontal cortex. Their findings showed that different cell clusters and specific oligodendrocyte subtypes, such as oligodendrocyte_C6, were involved in AD pathology. They also found elevated levels of PLXDC2, which may indicate its presence and hold potential for early AD detection.

Molecular predictors driven by RNA, including long non-coding RNAs (lncRNAs) and microRNAs (miRNAs), play an important role in AD [24]. The literature indicates that lncRNAs and miRNAs can affect messenger RNAs (mRNAs) and contribute to AD pathogenesis, making them promising targets for AD biomarker discovery [29,30]. A case in point is the work by Zhao et al. (2020) [31], which tackled the use of microRNA-based biomarkers in AD, and the significance of abnormal miRNA expression in the brain tissues and biofluids. The implementation of miRNAs’ diagnostic, prognostic, and therapeutic roles could open new avenues for future AD investigations, enhancing biomarker discovery and specificity. For a comprehensive understanding of gene biomarker discovery in AD, including the methods and identified genes, refer to [25], which provides an in-depth analysis of AD biomarkers.

This study presents an innovative multi-filter gene selection method designed to identify reliable biomarkers for Alzheimer’s disease (AD). Recognizing the complexity of AD and the urgent need for effective biomarkers and therapeutic targets, our approach integrates four complementary feature-ranking techniques: Random Forest (RF), Double Input Symmetrical Relevance (DISR), Degree Centrality, and Bottleneck Centrality. These methods were carefully selected to capture diverse aspects of gene relevance. While RF and DISR emphasize discriminative power—identifying genes with strong predictive value for AD by evaluating their ability to distinguish AD cases from normal ones—DISR further addresses pairwise feature dependencies to minimize redundancy. Meanwhile, Degree Centrality and Bottleneck Centrality, derived from the PPI network, assess the role of genes within the broader biological context. Degree Centrality identifies genes with the highest number of direct interactions, while Bottleneck Centrality highlights genes critical for maintaining network connectivity, potentially serving as regulatory hubs in AD pathophysiology. By integrating these four ranking methodologies, we guarantee a thorough assessment of gene significance from both predictive and network-based viewpoints, thereby augmenting the robustness and precision of biomarker identification for Alzheimer’s disease. We analyzed five publicly available AD-related microarray datasets, representing diverse brain regions and comprising a total of 803 DEGs from 464 AD cases and 492 normal cases. The top 50 genes were prioritized based on their aggregated rankings, and their predictive power was validated using an independent test dataset (GSE109887). Our findings provide valuable insights into potential therapeutic targets by revealing key biological processes involved in AD, such as neurodegeneration and cognitive function.

## 2. Results

### 2.1. Differentially Expressed Genes Analysis Across Individual AD Datasets

**Figure 1:**
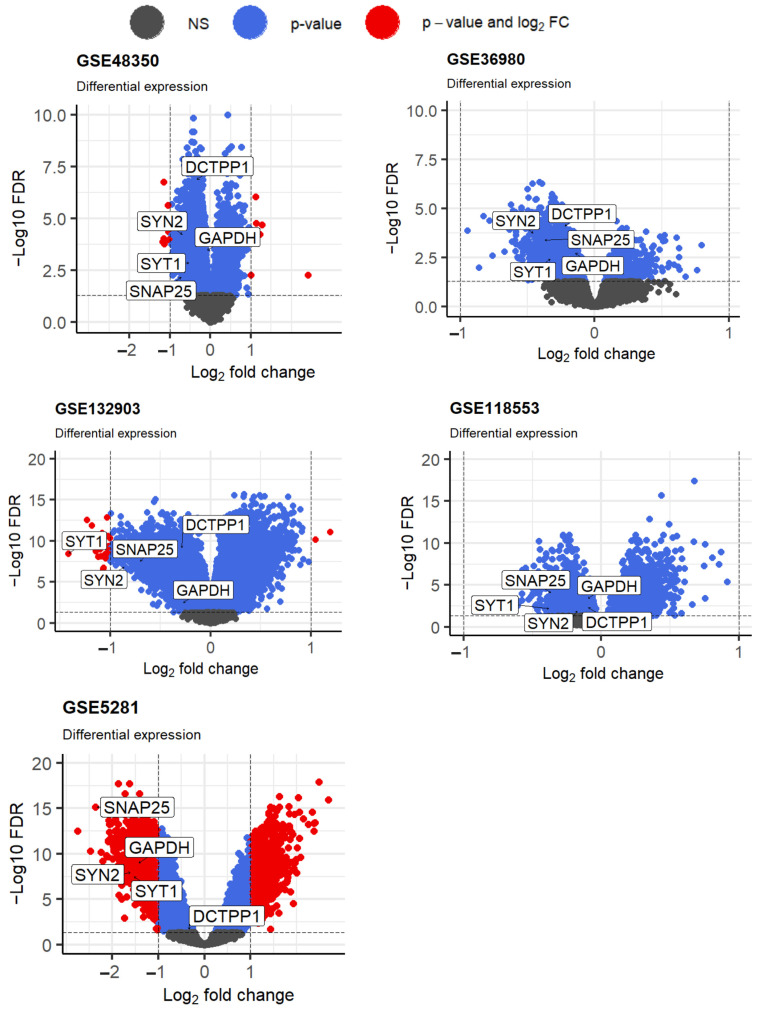
The volcano plot for DECs in AD is based on five microarray datasets from GEO. The top five genes selected by the proposed approach are shown on each plot.

We utilized the “limma” package (version 3.56.2) in R to identify DEGs from each of the five GEO datasets (GSE48350, GSE36980, GSE132903, GSE118553, and GSE5281). Using FDR< 0.05, we identified 9010 (up-regulated: 3117 vs. down-regulated: 5893), 4191 (up-regulated: 890 vs. down-regulated: 3265 DEGs), 11,773 (up-regulated: 6112 vs. down-regulated: 5661), 6516 (up-regulated: 3558 vs. down-regulated: 2958), and 10,003 (up-regulated: 4223; down-regulated: 5780) DEGs between AD and normal cases from GSE48350, GSE36980, GSE132903, GSE118553, and GSE5281 datasets, respectively, and their volcano plots are presented in Figure 1.

### 2.2. Identification and Analysis of Common DEGs Across Five AD Datasets

**Figure 2:**
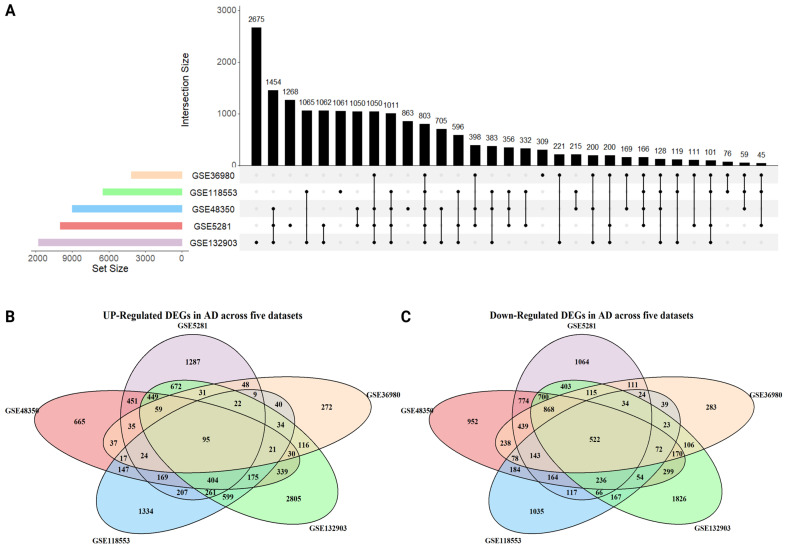
Overlap of DEGs across five Alzheimer’s disease datasets. (**A**) UpSet plot visualizing the overlap of DEGs across the datasets. (**B**) Overlap of upregulated DEGs shared among the five datasets. (**C**) Overlap of downregulated DEGs shared among the five datasets.

To identify common DEGs across datasets, we analyzed the overlap among GSE48350, GSE36980, GSE132903, GSE118553, and GSE5281. A total of 803 overlapping genes were identified, as visualized using an UpSet plot in Figure 2A. Among these, 95 genes were consistently upregulated, and 522 genes were consistently downregulated across all five datasets (Figure 2B,C: Venn diagrams). Additionally, 186 genes exhibited variable expression patterns, showing differing behaviors among the datasets.

### 2.3. PPI Network Construction and Ranking of Central Hub Genes

The STRING database was used to construct a PPI network, revealing significant connections between proteins encoded by the common DEGs. To identify hub genes within the network, two algorithms—degree of connectivity and bottleneck—were applied using the “igraph” package (version 2.0.3) in R. Genes were ranked separately by each algorithm, and the ranks were aggregated to generate a comprehensive list. From the aggregated ranking, the top 100 hub genes were selected for further analysis. To evaluate the predictive power of the identified hub genes, we used four classification algorithms: Logistic Regression (LR), Support Vector Machine (SVM) with a linear kernel, BayesNet, and Naive Bayes (NB). Table 1 presents the AUC scores for each classifier, evaluated on the independent test set GSE109887. The performance of classifiers was assessed across gene subsets ranging from the top 10 to the top 100 ranked genes, using aggregated PPI topological features (Degree and Bottleneck).

### 2.4. AUC Performance of the Proposed Multi-Filter Gene Selection Approach

**Figure 3:**
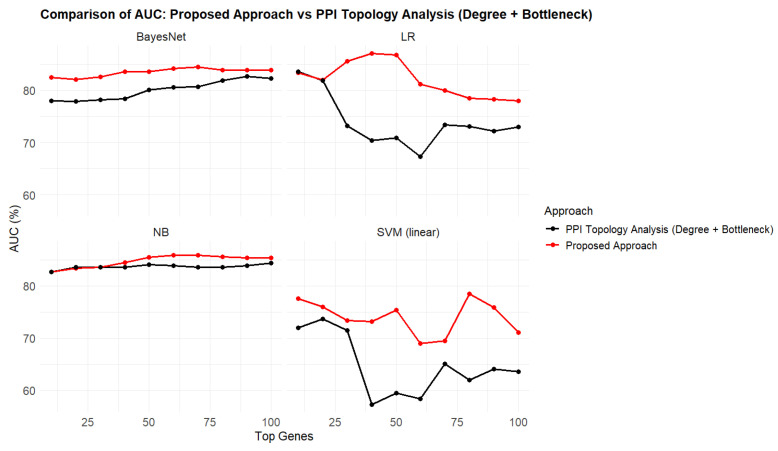
Comparative AUC performance of the proposed multi-filter approach and the commonly used PPI topology analysis on GSE109887 test data. The figure illustrates the AUC performance of four classification algorithms (Logistic Regression, SVM, BayesNet, and Naive Bayes) across the top 10 to the top 100 ranked genes. The proposed multi-filter approach (red) consistently outperforms the PPI Topology Analysis (black), which aggregates degree and bottleneck metrics for gene ranking.

The proposed multi-filter gene selection approach combines RF ranking, DISR filtering, and topological network analysis (using Degree and Bottleneck measures) to rank 803 overlapping genes found in five AD datasets. Table 2 shows the AUC scores for different classification algorithms (LR, SVM, BayesNet, and NB) across top-ranked gene subsets (top 10 to top 100), with the background color highlighting the highest AUC for each classifier. The top 40 genes selected by this method achieved the best average AUC values, with LR reaching 87.1%. NB ranked second, performing slightly lower at 85.9% when evaluated on the top 60 genes. Figure 3 illustrates a performance comparison between the proposed multi-filter method and the widely used PPI Topology Analysis approach. The results show that the multi-filter method consistently outperformed PPI Topology Analysis across all classifiers. This demonstrates the effectiveness of the proposed method in identifying predictive gene subsets and enhancing classification accuracy.

### 2.5. Rationale for Selecting the Top 50 Genes

The selection of the top 50 genes was driven by their ability to balance classification accuracy and biological significance. This subset consistently achieved high AUC values across multiple classifiers, reflecting strong predictive performance. Pathway enrichment analysis highlighted their involvement in critical biological pathways, including the Synaptic Vesicle Cycle, Alzheimer’s Disease, and Neurodegeneration Pathways, underscoring their relevance to the phenotype studied. Focusing on the top 50 ensured sufficient representation of biologically meaningful patterns while minimizing overfitting and noise. This concise gene set provides a robust foundation for validation and downstream pathway analyses.

### 2.6. Gene Expression and Correlation Analysis of the Top 50 Genes Identified by the Proposed Approach

**Figure 4:**
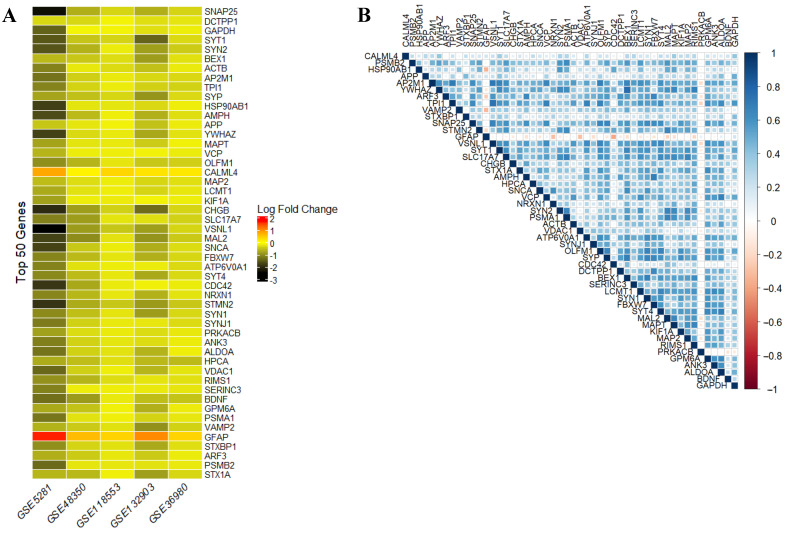
Log_2_fold change expression and correlation of top 50 genes across five AD datasets with 956 samples. (**A**) Heatmap showing the log_2_FC expression of the top 50 genes identified by the proposed multi-filter approach across five datasets, with a total of 956 samples. The top genes are represented in rows, and the datasets are represented in columns. The color scale ranges from blue (downregulated genes) to red (upregulated genes), with white indicating no significant change in expression. The dataset names are italicized, and gene clustering was applied. (**B**) Correlation matrix of the top 50 genes across the five datasets, with 956 samples, highlighting the relationships between gene expression patterns.

The expression analysis of the top 50 genes, visualized as a log_2_fold change heatmap (Figure 4A), reveals differential expression patterns across five AD datasets. Most genes were significantly downregulated, apart from “CALML4” and “GFAP”, which were upregulated. These expression patterns (whether upregulated or downregulated) highlight the relevance of these genes in AD progression. Additionally, the correlation matrix in Figure 4B demonstrates consistent relationships between the gene expression profiles, further supporting the robustness and biological coherence of the selected gene subset. Notable correlations include the following:

- BEX1 and YWHAZ: 0.7689, indicating a strong positive correlation.
- VSNL1 and TPI1: 0.736279, reflecting a moderate to strong positive correlation.
- AP2M1 and VSNL1: 0.715232, suggesting a moderate positive correlation.

Incorporating RF and DISR with topological network analysis (Degree and Bottleneck measures) led to the selection of biologically meaningful and functionally relevant gene sets. Using the top 50 features, our approach demonstrated superior classification accuracy on the independent test set across multiple classifiers. Furthermore, pathway enrichment analysis revealed significant involvement in key processes, including the Synaptic Vesicle Cycle, AD, and neurodegeneration pathways, highlighting the biological relevance of the identified genes.

### 2.7. Enrichment Analysis of the Top 50 Selected Genes

**Figure 5:**
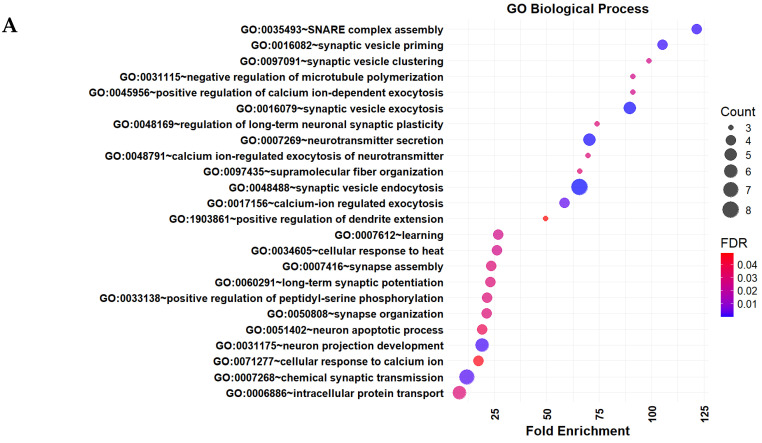

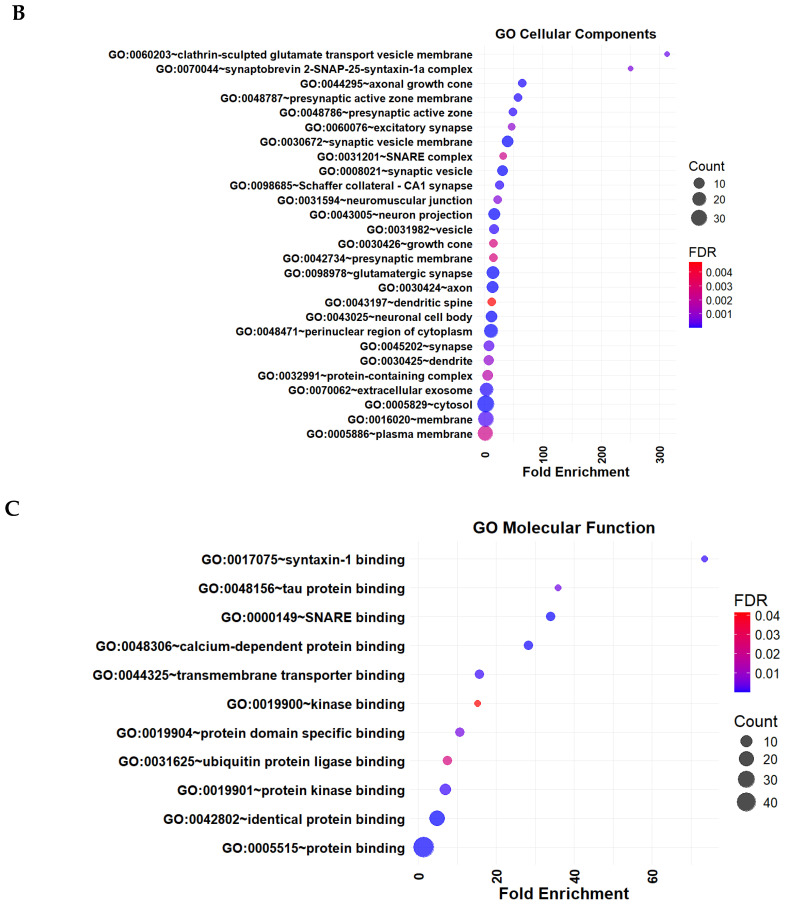

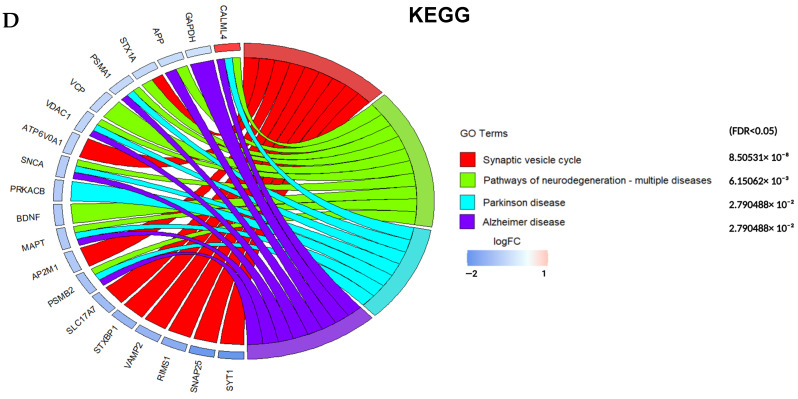
Gene Ontology (GO) enrichment annotations of the top 50 genes selected by the proposed approach: (**A**) Biological Process, (**B**) Cellular Component, (**C**) Molecular Function, (**D**) Significantly enriched Kyoto Encyclopedia of Genes and Genomes (KEGG) pathways with an FDR < 0.05. The results of the GO and the KEGG analyses were obtained from the ‘DAVID’ web tool (https://davidbioinformatics.nih.gov/tools.jsp) (accessed on 15 December 2024) and visualized by R package ‘ggplot2’ (version 3.5.1). The cohort plot illustrates the correlation of the top 50 genes with their assigned KEGG terms, represented by ribbons. The False Discovery Rate (FDR) was calculated using the Benjamini-Hochberg method to adjust for multiple hypothesis testing.

GO and KEGG pathway enrichment analyses were carried out on the top 50 ranked genes to explore their functional roles. GO analysis mapped the genes into three primary categories: Biological Process (BP), Cellular Component (CC), and Molecular Function (MF). Significant terms were identified based on an FDR threshold of less than 0.05. In this study, these genes were found to be significantly associated with various biological processes implicated in AD pathogenesis, including synaptic vesicle activities, neurotransmitter secretion, synaptic plasticity, learning and memory, calcium regulation, and neuronal apoptosis (Figure 5A). All these processes play an important role in synaptic function, neurotransmission, and neuronal survival in the neurodegenerative features of AD. Table 3 presents the significantly enriched KEGG pathways and GO terms of the top 50 genes, providing further insights into their involvement in critical biological processes. For example, Table 3 illustrates that SNCA, APP, and SYNJ1 are significantly implicated in synaptic and neurodegenerative processes, underscoring their probable roles in Alzheimer’s disease pathology. Additionally, SNAP25, STX1A, and NRXN1 are identified as playing crucial roles in synaptic vesicle function, which is essential for neurotransmission and synaptic plasticity. Moreover, GAPDH is significantly associated with neuronal apoptosis, a key feature of AD progression.

Furthermore, in terms of cellular components, the genes were enriched in regions vital for synaptic function and neuronal connectivity, such as synaptic vesicles, pre- and postsynaptic regions, dendritic spines, excitatory synapses, and neuronal projections (Figure 5B). This highlights their importance in AD-related disruptions of neural connectivity. MF analysis revealed the important molecular activities attributed to tau protein binding, SNARE binding, syntaxin-1 binding, ubiquitin protein ligase binding, and calcium-dependent protein binding in AD pathophysiology (Figure 5C). These are associated with processes such as tau aggregation, synaptic dysfunction, impaired protein degradation, and dysregulated calcium signaling. KEGG pathway analysis further confirmed that the top 50 genes were significantly enriched in key neurodegenerative pathways, including the synaptic vesicle cycle, neurodegenerative pathways, Parkinson’s disease, and Alzheimer’s disease (Figure 5D). Several downregulated genes, such as APP, MAPT, SNCA, VDAC1, BDNF, SNAP25, GAPDH, and SYT1 were implicated in AD-related pathological processes, including amyloid plaques, tau tangles, mitochondrial dysfunction, synaptic abnormalities, and impaired protein degradation. These findings suggest the important roles of these genes in AD pathogenesis and provide valuable insights that might help in future therapeutic research.

**Figure 6:**
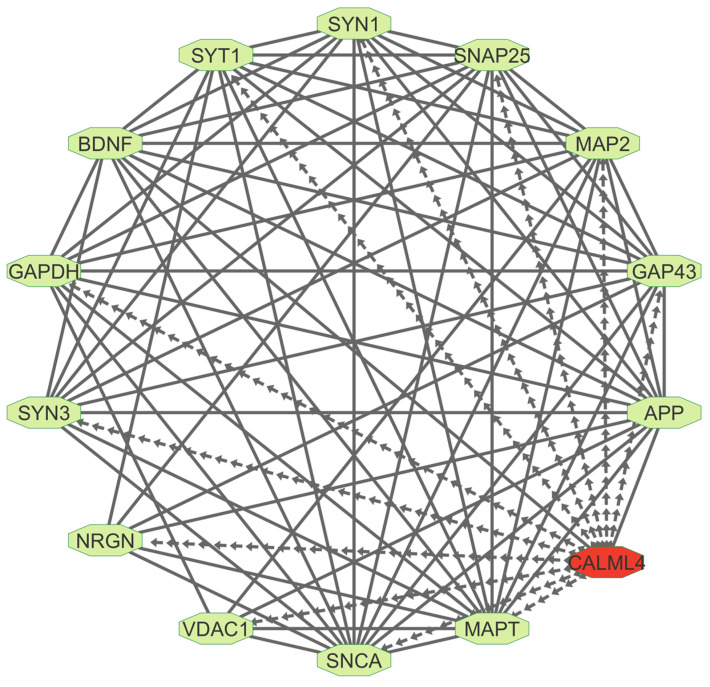
Protein–Protein interaction network of CALML4 and Alzheimer’s disease-related genes. The network shows interactions between CALML4 (red) and established AD-related genes. Dotted edges represent CALML4’s connections to AD-associated proteins, while solid edges indicate known interactions among the other genes.

Beyond established biomarkers, we identify CALML4 (calmodulin-like 4) as a potential novel biomarker for Alzheimer’s disease (AD). While Calmodulin (CaM), a calcium-binding protein, has been implicated in AD, CALML4 remains largely uncharacterized. Here, we show that CALML4 is enriched in neurodegenerative pathways, including AD (hsa05010), Parkinson’s disease (hsa05012), and neurotrophin signaling (hsa04722). Its interactions with key AD biomarkers, including APP, MAPT, SNAP25, GAPDH, and SNCA (Figure 6), suggest a potential role in AD pathogenesis.

## 3. Discussion

Identification of crucial genes in AD is essential for advancing research into the disease’s pathophysiology and identifying potential therapeutic targets. Our multi-filter gene selection approach identified a set of genes highly relevant to AD progression, based on their expression patterns and involvement in key biological processes. Several genes of particular interest were highlighted in this study. APP, ranked 13th, is significantly implicated in AD due to its role in amyloid-β formation during neuronal growth and maturation. Aβ plaques represent one of the major neuropathological features of AD, and the involvement of APP in their formation builds it as a major target for therapeutic intervention [32]. MAPT, which encodes tau and is ranked 15th in the analysis, is yet another important gene linked to neurofibrillary tangles—one more hallmark of AD. Mutations in MAPT have been shown to cause tauopathies, reinforcing tau’s critical role in AD pathology [33].

The interactions with amyloid-β and tau add to the progression of the disease itself, making it an important biomarker for cognitive decline in AD patients [34]. Voltage-Dependent Anion Channel 1 (VDAC1), ranked 39th, participates in mitochondrial function and has been implicated in playing a role in oxidative stress and apoptosis in AD. Its interaction with amyloid-β and tau further supports its potential as a therapeutic target [35]. Additionally, Brain-Derived Neurotrophic Factor (BDNF), ranked 42nd, is vital for maintaining synaptic plasticity, which is crucial for learning and memory. Impairment in BDNF signaling is closely linked to AD, as it is associated with tau phosphorylation and amyloid-β accumulation, highlighting its potential as a therapeutic target to slow disease progression [36]. SNAP25, ranked 1st, is a key presynaptic protein. Its increased levels in the CSF of AD patients, even at the early stage, suggest its potential as a biomarker for synaptic degeneration and AD progression [37]. GAPDH, ranked 3rd, forms toxic amyloid-β aggregates that contribute to neuronal damage, further supporting its role as a therapeutic target [38]. The 4th-ranking gene, SYT1, plays a crucial role in synaptic function and is elevated in the CSF of AD patients, where its synaptic dysfunction may be significant. This further supports its potential as a diagnostic and monitoring biomarker for the disease [39]. Application of the multi-filter approach with RF ranking, DISR filtering, and topological network analysis identified this set of genes, with high AUC scores resulting from classification models. With the selection of the top 50 genes, which showed very good predictive performance but still had some degree of biological relevance, this approach balances accuracy and biological insight and forms a robust method for gene selection in AD studies. Moreover, pathway enrichment analysis indicated that these genes were associated with several AD-related critical biological processes and pathways such as the Synaptic Vesicle Cycle, Neurodegeneration, and AD pathways.

SNAP25, SYN2, SYP, SYT1, APP, SNCA, BDNF, GFAP, VAMP2, MAPT, and SLC17A7 are among the top 50 genes identified as potential targets for early-stage AD [40,41,42,43,44,45,46]. SYT1 (Synaptotagmin 1) is a key protein involved in synaptic function, and its dysfunction could contribute to early synaptic changes in AD. miR-137 and miR-146a are reported to target SYT1, suggesting its importance in modulating early AD-related synaptic processes [47].

Beyond these well-established biomarkers, our study has identified CALML4 (calmodulin-like 4) as a promising novel biomarker for AD. While Calmodulin (CaM) has been implicated in AD due to its calcium-binding properties, CALML4, a closely related protein, has not been extensively explored in this context. Pathway enrichment analysis shows that CALML4 is enriched in neurodegenerative pathways, including those associated with AD, Parkinson’s disease, and neurotrophin signaling. Additionally, CALML4 interacts with key AD biomarkers such as APP, MAPT, SNAP25, GAPDH, and SNCA, suggesting a potential role in AD pathogenesis. Its involvement in critical biological pathways further strengthens its candidacy as a target for future therapeutic development.

These findings thus provide important clues regarding the molecular mechanisms behind AD and potential targets for therapeutic intervention. The top 50 genes, identified in this work and related to synaptic function, calcium regulation, and neuronal survival, will provide a firm basis for future research into the causes of AD and the development of its treatment. Nevertheless, there are significant restrictions to acknowledge. First, the absence of experimental validation remains a key constraint, as the identified biomarkers, despite their strong predictive value, have not been confirmed in vitro or in vivo. Second, reliance on publicly available microarray datasets introduces potential biases due to variations in data quality, sample sizes, and experimental conditions. Third, the lack of external clinical validation further limits the generalizability of our findings.

## 4. Materials and Methods

### 4.1. Data Acquisition and Preprocessing

This study used five publicly available gene expression datasets from Deoxyribonucleic Acid (DNA) microarray analyses of human brain tissues. These datasets were sourced from the NCBI Gene Expression Omnibus (NCBI-GEO) database (https://www.ncbi.nlm.nih.gov/geo/) (accessed on 15 November 2024). The datasets, including GSE5281, GSE118553, GSE132903, GSE48350, and GSE36980, were chosen for their insights into brain regions relevant to AD. Various brain regions, such as the medial temporal gyrus (MTG), hippocampus (HIP), entorhinal cortex (EC), and others, were represented, with varying numbers of cases analyzed. Each dataset was analyzed separately to identify DEGs associated with AD, and common DEGs were identified across the datasets. A summary of the key characteristics of each dataset, including brain regions, the number of genes, and the distribution of AD and normal cases, is provided in Table 4.

### 4.2. Identification of DEGs from Each Dataset

To identify DEGs between AD and normal cases, each selected dataset was analyzed using the “limma” package in R (version 3.56.2). The log_2_FC and adjusted *p*-values of each gene were calculated. The “annotation” package (version 1.30.0) from Bioconductor was used to convert microarray probe data into gene symbols. When multiple probes were mapped to the same gene symbol, the gene with the lowest associated adjusted *p*-value was selected. DEGs between AD and normal cases were identified with a significance threshold of false discovery rate (FDR) < 0.05 (adjusted *p*-value using the Benjamini-Hochberg method). A volcano plot of the DEGs was generated using the “ggplot2” package (version 3.5.1) in R.

### 4.3. Identification of Common DEGs

After identifying DEGs using the “limma” package, we determined the shared or overlapping DEGs across the five datasets using the following formula:

(1) $$\mathrm{Common}\;\mathrm{DEGs}=i={⋂}_{i=1}^{r}{Identified\;DEGs\;from\;GEO\;dataset}_{i}$$

where r is the number of datasets used (in this case, r=5). For visualization, a Venn diagram and an intersection plot of DEGs were generated using the “VennDiagram” package (version 1.7.3) in R. Additionally, an UpSet plot was created using the “UpSetR” package (version 1.4.0).

### 4.4. PPI Network Construction and Hub Gene Prioritization

A PPI network was constructed using the STRING database (https://www.string-db.org/) (accessed on 10 December 2024) to elucidate the functional relationships among the 803 common DEGs. The analysis was performed with a minimum confidence interaction score of 0.4 to ensure the inclusion of statistically robust interactions. The resulting interaction data were imported into R and analyzed using the “igraph” package to evaluate the network’s topological properties. Key centrality measures, including degree centrality (quantifying the number of direct connections of each node) and bottleneck centrality (identifying nodes critical for maintaining network connectivity), were calculated. These metrics were chosen for their balance between computational efficiency and biological relevance. By ranking genes based on their connectivity and centrality within the network, this analysis enabled the identification of hub genes, which are likely to serve as key regulators in the underlying molecular mechanisms.

### 4.5. Feature Ranking Using RF and DISR

The combined dataset, comprising 464 AD cases and 492 normal controls with 803 common genes, was analyzed to identify the most informative genes. To identify the most informative genes for further analysis, two complementary approaches were employed: RF ranking and Double Input Symmetrical Relevance (DISR). RF, a robust ensemble learning algorithm, was utilized to rank features based on their importance in classification tasks. Feature importance scores were derived by assessing the reduction in impurity (quantified using the Gini index or entropy) across decision trees within the forest. Features with higher importance scores were considered more predictive, facilitating the identification of key variables with strong discriminative power. The analysis was implemented using the “randomForest” package (version 4.7.1.2) in R. DISR is a mutual information-based metric designed to measure the symmetrical relevance of features with respect to the target class while accounting for pairwise feature dependencies. Unlike conventional mutual information methods, DISR reduces redundancy by incorporating feature interactions, thereby identifying features that are both highly informative and non-redundant. The analysis was done using the “FSelector” package (version 0.34) in R. By combining these approaches, the analysis balanced predictive power and relevance, ensuring the selection of a biologically meaningful feature set. RF emphasized features’ predictive capability, while DISR complemented this by prioritizing non-redundant and highly relevant variables. Together, these methods facilitated a comprehensive identification of the most discriminative genes.

### 4.6. Proposed Multi-Filter Gene Selection Approach

To enhance gene selection, we proposed a multi-filter approach that integrates four feature ranking methods: RF, DISR, Degree Centrality, and Bottleneck Centrality.

RF and DISR were applied to an expression matrix combining five AD studies, evaluating genes based on their ability to differentiate AD from normal cases. RF assesses the predictive power of individual genes, while DISR captures the relevance of genes by considering pairwise feature dependencies and reducing redundancy.

To incorporate biological significance, Degree and Bottleneck Centralities were computed from the PPI network. Degree Centrality identifies genes with the highest number of direct interactions, potentially indicating key players in the molecular network. In contrast, Bottleneck Centrality highlights genes critical for maintaining network connectivity, pointing to regulatory hubs that may be essential for AD pathophysiology.

**Figure 7:**
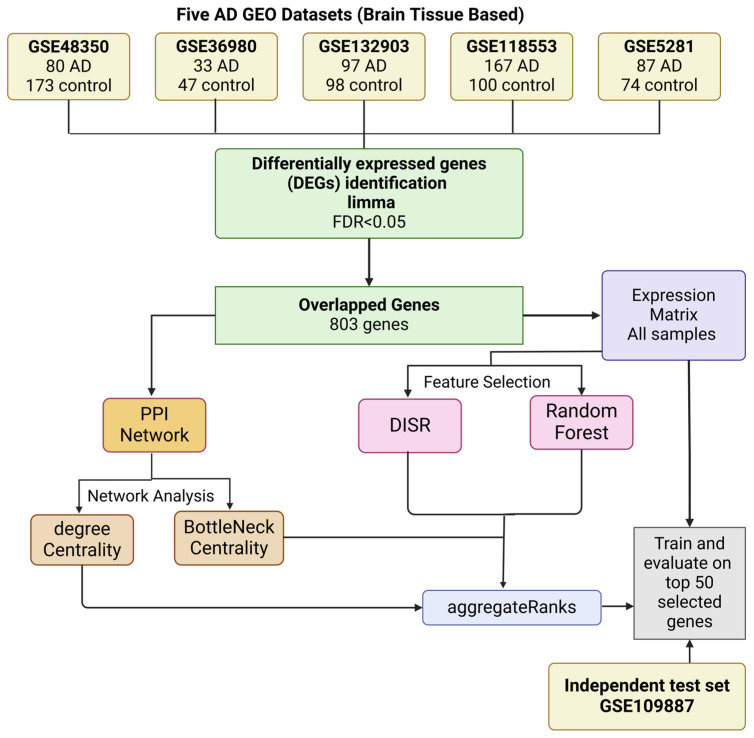
Workflow of the multi-filter gene selection approach. This workflow integrates cross-platform analysis to identify common genes, incorporates network analysis, and employs gene ranking techniques using DISR and Random Forest. The aggregated results are used to identify the top 50 genes.

All four ranking methods were applied in parallel, generating separate ranked lists of genes. These rankings were then aggregated using the “RobustRankAggreg” package (version 1.2.1) in R, which assigns a consensus score based on the relative ranks of genes across different methods. This approach ensures that the selected genes are not only statistically relevant to AD but also biologically meaningful within a molecular network context. Figure 7 illustrates the workflow of the multi-filter gene selection approach.

Based on these aggregated ranks, the top 100 genes were evaluated for predictive performance using an independent test dataset, GSE109887. The predictive performance of the selected genes was assessed with four classifiers: LR, SVM with a linear kernel, BayesNet, and NB. Genes ranked within the top 50 demonstrated superior performance across these classifiers, as indicated by AUC metrics, underscoring their relevance for downstream analyses.

### 4.7. Expression Analysis of the Top 50 Genes in AD

The expression profiles of the top 50 genes identified by the multi-filter gene selection approach were systematically analyzed to assess their differential expression and inter-gene correlations across multiple AD datasets. To visualize the log_2_FC in gene expression across the selected datasets, the “pheatmap” package (version 1.0.12) in R was utilized. This heatmap allowed for the representation of gene expression variations, where rows corresponded to the top 50 genes and columns to the datasets. To construct a correlation matrix for the top 50 genes and highlight the relationships between their expression patterns across the datasets, the “corrplot” package (version 0.92) in R was used. The correlation coefficients were computed to quantify the strength of the association between pairs of genes, offering insights into the degree of similarity or divergence in gene expression profiles.

### 4.8. Enrichment Analysis of Top 50 Genes

To gain deeper insights into the mechanisms and progression of AD, we performed enrichment analysis, including Gene Ontology (GO) and Kyoto Encyclopedia of Genes and Genomes (KEGG) pathway analyses, on the top 50 selected genes. This analysis used the DAVID (Database for Annotation, Visualization, and Integrated Discovery) version 6.8 tool. An FDR threshold of <0.05 was applied to identify significant enrichment results, ensuring the reliability of the findings.

## 5. Conclusions

Extensive research spanning decades has focused on elucidating the mechanisms of AD, yet a definitive solution remains elusive. Recently, efforts have shifted towards identifying new therapeutic targets, as previous drug development failures were largely attributed to inefficient target selection. Advances in high-throughput technologies have also increased data availability, facilitating the development of classifiers that could serve as decision-support tools for clinicians. This research illustrates that the suggested multi-filter gene selection method can pinpoint highly predictive gene subsets linked to AD. By aggregating the rankings of several methods, we prioritized 50 genes out of the 803 overlapping genes in five datasets and demonstrated their strong predictive power with AUC  =  0.87. These genes are involved in critical pathways related to AD and present promising candidates for further investigation and potential therapeutic targeting. This approach opens the possibilities of more effective gene-based strategies in both research and therapy for AD by fitting computational methods together with biological relevance.

## Figures and Tables

**Table 1:** AUC performance of PPI topology analysis (degree + bottleneck) on GSE109887 test data.

Classifiers	Top 10	Top 20	Top 30	Top 40	Top 50	Top 60	Top 70	Top 80	Top 90	Top 100
LR	83.6	81.9	73.2	70.4	70.9	67.3	73.4	73.1	72.2	73
SVM (linear)	72	73.7	71.5	57.3	59.5	58.4	65.1	62	64.1	63.6
BayesNet	78	77.9	78.2	78.4	80.1	80.6	80.7	81.9	82.7	82.3
NB	82.7	83.6	83.6	83.6	84.1	83.9	83.6	83.6	83.9	84.4

**Table 2:** AUC performance of the proposed multi-filter gene selection approach on GSE109887 test data. The background color highlights the highest AUC for each classifier across different gene set sizes.

Classifiers	Top 10	Top 20	Top 30	Top 40	Top 50	Top 60	Top 70	Top 80	Top 90	Top 100
LR	83.4	82	85.6	87.1	86.8	81.2	80	78.5	78.3	78
SVM (linear)	77.6	76	73.4	73.2	75.4	69	69.5	78.5	75.9	71.1
BayesNet	82.5	82.1	82.6	83.6	83.6	84.2	84.5	83.9	83.9	83.9
NB	82.7	83.4	83.6	84.5	85.5	85.9	85.9	85.6	85.4	85.4

**Table 3:** Significantly enriched KEGG pathway and GO terms of the top 50 genes.

Category	Term	Genes	FDR
KEGG			
KEGG_PATHWAY	hsa04721:Synaptic vesicle cycle	RIMS1, SNAP25, SYT1, STXBP1, SLC17A7, STX1A, AP2M1, VAMP2, ATP6V0A1	8.50531 × 10^−^⁸
KEGG_PATHWAY	hsa05022:Pathways of neurodegeneration— multiple diseases	APP, VCP, PSMA1, PSMB2, BDNF, VDAC1, CALML4, MAPT, STX1A, SNCA	6.15062 × 10^−^³
KEGG_PATHWAY	hsa05012:Parkinson disease	PSMA1, PSMB2, VDAC1, CALML4, MAPT, PRKACB, SNCA	2.790488 × 10^−^²
KEGG_PATHWAY	hsa05010:Alzheimer disease	APP, PSMA1, PSMB2, VDAC1, CALML4, MAPT, GAPDH, SNCA	2.790488 × 10^−^²
GO terms			
GOTERM_BP_DIRECT	GO:0048488~synaptic vesicle endocytosis	SYNJ1, SYT1, AMPH, SYP, STX1A, AP2M1, VAMP2, SNCA	1.838899 × 10^−^⁸
GOTERM_BP_DIRECT	GO:0016079~synaptic vesicle exocytosis	RIMS1, SNAP25, STX1A, VAMP2, SNCA	8.606257 × 10^−^⁵
GOTERM_BP_DIRECT	GO:0016082~synaptic vesicle priming	SNAP25, SYNJ1, STXBP1, SNCA	9.294342 × 10^−^⁴
GOTERM_BP_DIRECT	GO:0007269~neurotransmitter secretion	SYT1, NRXN1, STXBP1, SYN2, SYN1	0.0001588463
GOTERM_BP_DIRECT	GO:0097091~synaptic vesicle clustering	NRXN1, SYN2, SYN1	2.581858 × 10^−^²
GOTERM_BP_DIRECT	GO:0007612~learning	APP, SYNJ1, NRXN1, VDAC1	0.02581858
GOTERM_BP_DIRECT	GO:0048791~calcium ion-regulated exocytosis of neurotransmitter	RIMS1, SYT4, SYT1	0.02986648
GOTERM_BP_DIRECT	GO:0048169~regulation of long-term neuronal synaptic plasticity	APP, SYP, SNCA	0.02986648
GOTERM_BP_DIRECT	GO:0051402~neuron apoptotic process	APP, STXBP1, GAPDH, SNCA	0.03658041
GOTERM_CC_DIRECT	GO:0043005~neuron projection	ARF3, GPM6A, SNAP25, SYT1, STMN2, ANK3, SYP, CDC42, MAP2, SLC17A7, MAPT, STX1A, VAMP2	5.677075 × 10^−^¹⁰
GOTERM_CC_DIRECT	GO:0008021~synaptic vesicle	SNAP25, APP, SYT1, BDNF, AMPH, SYP, STX1A, SYN1, VAMP2	1.587475 × 10^−^⁸
GOTERM_CC_DIRECT	GO:0060076~excitatory synapse	SYT1, NRXN1, SLC17A7, SYP	9.228951 × 10^−^⁴
GOTERM_CC_DIRECT	GO:0098794~postsynapse	SNAP25, STXBP1, AP2M1, SNCA	3.265619 × 10^−^²
GOTERM_CC_DIRECT	GO:0098793~presynapse	SYNJ1, NRXN1, SYN1, ACTB	4.388568 × 10^−^²
GOTERM_CC_DIRECT	GO:0043197~dendritic spine	CDC42, GPM6A, APP, MAPT, PRKACB	0.004691407
GOTERM_MF_DIRECT	GO:0000149~SNARE binding	SYT4, SYT1, STXBP1, STX1A, VAMP2, SNCA	5.396982 × 10^−^⁵
GOTERM_MF_DIRECT	GO:0048306~calcium-dependent protein binding	SNAP25, SYT1, NRXN1, STMN2, SYN1, VAMP2	0.0001031687
GOTERM_MF_DIRECT	GO:0017075~syntaxin-1 binding	SNAP25, SYT1, STXBP1, VAMP2	0.0008115585
GOTERM_MF_DIRECT	GO:0048156~tau protein binding	HSP90AB1, MAP2, ACTB, SNCA	0.004525259
GOTERM_MF_DIRECT	GO:0031625~ubiquitin protein ligase binding	VCP, HSP90AB1, TPI1, FBXW7, YWHAZ, PRKACB	0.02299389

CC: cellular component; BP: biological process; MF: molecular function.

**Table 4:** Summary of gene expression datasets used in this study.

Dataset ID	Ref.	Platforms	Brain Regions	No. of Genes	AD Cases	Normal Cases	Total Cases	Use
GSE48350	[48]	GPL570	Entorhinal cortex, Hippocampus, Superior frontal gyrus, Postcentral gyrus	54,675	80	173	253	Train
GSE36980	[49]	GPL6244	Hippocampus, Temporal cortex, Frontal cortex	33,297	33	47	80	Train
GSE132903	[50]	GPL10558	Medial temporal gyrus	42,179	97	98	195	Train
GSE118553	[51]	GPL10558	Entorhinal cortex, Temporal cortex, Frontal cortex, Cingulate cortex	47,323	167	100	267	Train
GSE5281	[52]	GPL570	Hippocampus, Entorhinal cortex, Medial temporal gyrus, Posterior cingulate, Superior frontal gyrus, Primary visual cortex	54,675	87	74	161	Train
GSE109887	[53]	GPL10904	Medial temporal gyrus	31,700	46	32	78	Test

## Data Availability

The datasets generated and/or analyzed during the current study are available in the Gene Expression Omnibus (GEO) repository under the following accession numbers: GSE48350, GSE36980, GSE132903, GSE118553, GSE5281, and GSE109887. These datasets utilize the following platforms: GPL570, GPL6244, GPL10558, GPL10558, GPL570, and GPL10904. The datasets can be accessed and downloaded from the GEO website at www.ncbi.nlm.nih.gov/geo/ (accessed on 15 November 2024).
